# Bioactivity and Bioavailability of Carotenoids Applied in Human Health: Technological Advances and Innovation

**DOI:** 10.3390/ijms25147603

**Published:** 2024-07-11

**Authors:** Tomas Gabriel Bas

**Affiliations:** Escuela de Ciencias Empresariales, Universidad Catolica del Norte, Coquimbo 1780000, Chile; tomas.bas@ucn.cl

**Keywords:** carotenoids, advanced bioavailability, bioactivity, supramolecular carriers, personalized medicine, chronic disease prevention, extraction

## Abstract

This article presents a groundbreaking perspective on carotenoids, focusing on their innovative applications and transformative potential in human health and medicine. Research jointly delves deeper into the bioactivity and bioavailability of carotenoids, revealing therapeutic uses and technological advances that have the potential to revolutionize medical treatments. We explore pioneering therapeutic applications in which carotenoids are used to treat chronic diseases such as cancer, cardiovascular disease, and age-related macular degeneration, offering novel protective mechanisms and innovative therapeutic benefits. Our study also shows cutting-edge technological innovations in carotenoid extraction and bioavailability, including the development of supramolecular carriers and advanced nanotechnology, which dramatically improve the absorption and efficacy of these compounds. These technological advances not only ensure consistent quality but also tailor carotenoid therapies to each patient’s health needs, paving the way for personalized medicine. By integrating the latest scientific discoveries and innovative techniques, this research provides a prospective perspective on the clinical applications of carotenoids, establishing a new benchmark for future studies in this field. Our findings underscore the importance of optimizing carotenoid extraction, administration, bioactivity, and bioavailability methods to develop more effective, targeted, and personalized treatments, thus offering visionary insight into their potential in modern medical practices.

## 1. Introduction

Carotenoids comprise a group of fat-soluble organic pigments synthesized by phototrophs (algae, cyanobacteria, and plants), non-photosynthetic bacteria, and fungi, with more than 1117 structures identified from 683 different sources within the hydrocarbon class [1]. These pigments exhibit highly attractive biological activities, such as antioxidant properties, anti-inflammatory effects, and the attributes of provitamin A, making them attractive for applications in medicine and the therapeutic treatment of specific diseases [2,3,4,5,6]. Carotenoids can be classified into two main categories according to their chemical and nutritional characteristics: carotenes and xanthophylls [7,8,9,10]. Carotenes are characterized by the presence of carbon and hydrogen atoms in their chemical structure and include β-carotene, α-carotene, γ-carotene, δ-carotene, lycopene, neurosporene, and torulene, known for their provitamin content. Meanwhile, the xanthophyll category mainly comprises astaxanthin, zeaxanthin, lutein, torularhodin, canthaxanthin, and violaxanthin [11]. Carotenoids, as vital secondary metabolites, play a crucial role in maintaining various functions of human organs, including eye, cardiovascular, and skin health, immune function, cognitive function and bone health. Its mechanisms of action involve neutralizing free radicals, modulating gene expression, and regulating inflammatory pathways [12,13,14]. The structure of these various phytochemicals manifests itself as tetraterpenes with eight five-carbon isoprenoid units (mainly C40), containing up to 15 conjugated double bonds and exhibiting a symmetric arrangement typified by β-carotene, resulting in red, yellow, and oranges [11]. Natural sources of carotenoids are diverse and significant amounts are found in brown algae and microalgae such as *Heterochlorella luteoviridis*, *Dunaliella salina* or *Chlorella vulgaris*, by-products of carrot juice and skin processing, tomatoes, corn gluten meal, citrus residue, pomegranate residue, red pepper, *Lycium*, *ferocissimum fruits*, guava pulp, the peel and pulp of thai gac fruit (*Momordica cochinchinensis Spreng*), apricot waste, orange and pumpkin peels, vegetables, shrimp and yeast such as *Rhodotorula glutinis*, *Xanthophyllomyces* or *Dendrorhous*, among others [15,16]. These abundant sources offer promising prospects for the extraction of valuable carotenoids that are applicable not only in traditional industries such as food dyes, cosmetics, and nutraceuticals, but also in the pharmaceutical industry for human health [17,18,19,20].

The objective of this research is to document advances related to cutting-edge techniques in the extraction of carotenoids, highlighting the impact of their use in medicine and the pharmaceutical industry. This article features the most innovative methodologies and technological advances related to carotenoid extraction, with special focus on improving their bioactivity and bioavailability for applications in human health. The transformative potential of these cutting-edge extraction techniques is highlighted by detailing how they improve the efficiency, sustainability, and quality of carotenoid extraction. By emphasizing the scientific rigor and novelty of these methods, this article sets a new benchmark for future research and practical applications in the fields of food, pharmaceuticals, and nutraceuticals. This article addresses the bioactivity of carotenoids in the biopharmaceutical sector, seeking to understand the therapeutic and innovative implications of their administration and bioavailability in medicine. Different scientific results are analyzed that seek to provide a more comprehensive understanding of the fundamental role that these molecules can play in the medical treatment and management of certain specific diseases such as cancer, heart and eye diseases (macula), or inflammatory ailments.

## 2. The Functionality of Carotenoids

Carotenoids participate in different biological functions in plant, aquatic, animal and human systems [21]. In plants, plastids regulate biosynthesis, improving light absorption. In nonphotosynthetic tissues, carotenoids act as dyes and precursors of isoprene [11]. In human cells, they contribute to skin health, improving defense against ultraviolet (UV) radiation and also providing protection against diabetes, cancer, inflammation, and quality of vision [22,23,24]. Recently, research has been conducted on sources of so-called unusual colorless carotenoids, such as phytoene and phytofluene from aquatic environments, such as the microalgae *D. salina* [25,26]. These compounds are part of human plasma, internal tissues, and skin and are considered a sustainable endogenous nutrient to maintain healthy skin and prevent the symptoms of photoaging, the main source of skin aging [27]. Similarly, studies recognize the importance of apocarotenoids and their possible involvement in biological actions against cancer [28]. However, despite promising data, it is crucial to have reliable information on the content and volume of intake of different types of carotenoids for each specific case, to establish the recommended prescription units. This is key, since excessive administration of these compounds could cause undesirable and even dangerous side effects to the patient’s health [29,30].

Therefore, it is essential to know the molecular structure of carotenoids, because they determine their bioavailability, metabolism, and specific benefits for their use in medicine [31]. Table 1 shows the specificity of different molecular structures of seven carotenoids [32]. Recognizing these differences is essential to optimize their intake and take advantage of their potential in the prevention and health promotion.

Optimizing analytical methods at the molecular level involves several steps, including sample preparation, extraction, separation, detection, and quantification. This comprehensive approach to the distinction between different types of carotenoids and optimized analytical methods ensures accurate identification, quantification, and analysis of carotenoids at the molecular level. Table 2 shows a summary of the steps, samples, methods, and optimization.

### 2.1. Carotenoid Production Methods

Carotenoid production methods can be broadly classified into two types—synthetic and natural—although there are hybrid methods as well [33]. Synthetic carotenoids production is considerably faster and less expensive compared to natural carotenoids. However, the quality of synthetic carotenoids may not be the best option for developing molecules used in medicine and disease treatment [34,35]. In medicine, preference is shifting toward natural carotenoids over synthetic ones, as the latter can be harmful to human health in high doses and produce hazardous waste during manufacturing. However, carotenoids derived from natural compounds (plants, algae) are relatively more expensive to obtain, but offer complete traceability and safety of their components, crucial for human health [4].

#### Different Carotenoid Extraction Methods

The relentless pursuit of maximizing carotenoid extraction to increase recovery yields and reduce costs has led to numerous research efforts [36]. However, there is no single method that stands out above the rest. The method depends on the substrate used, the type of carotenoid sought, its final use (cosmetic, food or medicinal), and the level of purity required [12]. The choice of extraction method will depend on the different levels of polarity and susceptibility to oxidation of carotenoids, as well as the physical and chemical barriers of each carotenoid, which vary depending on the substrate and intended use [37]. Numerous studies address the diverse existing methods of extraction of carotenoid, from the most conventional to the most innovative [12]. In this range of innovative advances in carotenoid extraction methods, there has been a focus on enhancing yield, purity, and sustainability while minimizing environmental impact and ensuring the integrity of the bioactive compounds. We present a detailed description of cutting-edge techniques in carotenoid extraction, highlighting their scientific rigor and significance [36,37,38,39,40,41].

- SFE involves using carbon dioxide (CO_2_) above its critical temperature and pressure, where it exhibits the properties of both a liquid and a gas. This state allows CO_2_ to penetrate solid materials like a gas but dissolve substances like a liquid, making it highly effective for extracting carotenoids from plant matrices. Tunable parameters enable selective extraction, allowing for the targeting of specific carotenoids without the extraction of unwanted compounds. Recent innovations in SFE include the optimization of pressure and temperature parameters to improve carotenoid yield and selectivity. Integration with cosolvents such as ethanol has further enhanced the extraction efficiency for a broader range of carotenoids. This method is considered highly sustainable, as it eliminates the need for organic solvents, reduces the extraction time, and allows for easy separation and recycling of CO_2_. It is particularly valued for its ability to preserve the bioactivity of carotenoids and maintain their functional properties for subsequent applications.
- Pressurized liquid extraction (PLE), also known as accelerated solvent extraction (ASE), utilizes solvents at high temperatures and pressures to dissolve and extract carotenoids more effectively than traditional solvent extraction methods. Increased solubility and diffusivity of carotenoids under these conditions result in higher extraction efficiencies and shorter processing times. Innovations in PLE involve the use of environmentally friendly solvents such as water and ethanol under subcritical conditions, which reduce the environmental footprint and improve safety. Additionally, multistep gradient extraction techniques have been developed to selectively extract different carotenoids based on their polarity and solubility. PLE offers rapid extraction with higher yields and reduced solvent consumption, making it an efficient and sustainable alternative to conventional methods. The ability to fine-tune extraction parameters allows selective isolation of specific carotenoids, enhancing the purity and quality of the extracts.
- Ionic liquid extraction (ILE) uses ionic liquids, which are composed of ions, to extract carotenoids. These solvents offer a unique combination of low volatility, high thermal stability, and the ability to dissolve both polar and nonpolar compounds. The versatility and tunability of ionic liquids make them ideal for selective and efficient carotenoid extraction. The application of ionic liquids, which are salts in a liquid state, as green solvents for carotenoid extraction. ILE offers an eco-friendly use with ionic liquids that are often recyclable and less volatile than traditional solvents. It is possible to dissolve a wide range of compounds, including carotenoids. It can be tailored to target specific carotenoids.
- UAE uses ultrasound waves to create cavitation, which disrupts cell walls and facilitates the release of carotenoids into the solvent. Recent advances in UAE include the development of dual-frequency ultrasonic systems and the use of pulsed ultrasound, which improve extraction efficiency and reduce processing time. The combination of UAE with green solvents such as aqueous ethanol has further minimized the environmental impact. The UAE is recognized for its ability to enhance mass transfer, leading to higher carotenoid yields with lower energy consumption and shorter extraction times. The nonthermal nature of ultrasound preserves the chemical structure and bioactivity of carotenoids, making this method ideal for sensitive compounds.
- Microwave-assisted extraction (MAE) uses microwave energy to heat solvents and samples, causing rapid cell-wall disruption and the efficient release of carotenoids. Innovations in MAE include the use of ionic liquids and deep eutectic solvents, which offer superior solubilizing capabilities and enhance the extraction of carotenoids from complex matrices. The development of controlled microwave systems has enabled precise tuning of the energy input to optimize extraction conditions. MAE provides a rapid, efficient, and energy-saving approach to carotenoid extraction. The technique’s ability to target specific molecular interactions results in high yields and selectivity, preserving the integrity of carotenoids, and reducing the use of hazardous solvents.
- Enzyme-assisted extraction (EAE) uses specific enzymes to hydrolyze cell walls, facilitating the release of carotenoids into the extraction medium. Recent progress in EAE includes the identification and application of novel enzymes that specifically target cell wall components in different carotenoid sources. The combination of enzymes with mild extraction conditions has significantly improved the yield and purity of carotenoid extracts. EAE is particularly advantageous for its mild operating conditions, which preserve the bioactivity of carotenoids. The use of specific enzymes allows selective extraction, the minimization of the degradation of sensitive compounds, and improvements in the overall quality of the extract.
- Pulsed electric field (PEF) extraction involves the application of short pulses of high voltage to plant materials, creating temporary pores in cell membranes. This process, known as electroporation, enhances the permeability of cells, allowing for a more efficient release of carotenoids into the extraction solvent. PEF is particularly advantageous for extracting carotenoids from heat-sensitive materials. The development of advanced PEF systems with precise control over pulse parameters has enhanced the efficiency of extracting carotenoid from various matrices. The integration of PEF with subsequent extraction steps, such as solvent extraction, has further improved the carotenoid yield and purity. PEF is a nonthermal technique that preserves the structural integrity and bioactivity of carotenoids. It is considered to be environmentally friendly due to its low energy consumption and minimal use of solvents, making it an attractive option for sustainable carotenoid extraction.
- Hydrothermal extraction utilizes water at high temperatures and pressures to extract carotenoids, utilizing the solubilizing power of subcritical and supercritical water. Innovations in hydrothermal extraction include the use of continuous flow systems and the combination with supercritical CO_2_ to enhance extraction efficiency. The development of novel reactor designs has improved the scalability and application range of this technique. Hydrothermal extraction is valued for its use of water, a non-toxic and readily available solvent, making it an eco-friendly alternative. Its ability to operate under mild conditions ensures the preservation of carotenoid bioactivity and provides high extraction efficiency for both polar and non-polar carotenoids.

These advancements represent a significant leap in the efficiency, sustainability, and efficacy of carotenoid extraction processes. The integration of these cutting-edge techniques into industrial applications promises to enhance the availability and quality of carotenoid-rich products, supporting their use in health-promoting foods, supplements, and therapeutic agents. The scientific rigor behind these methodologies ensures that the integrity and bioactivity of carotenoids are maintained, maximizing their potential health benefits, and aligning with the growing demand for environmentally sustainable extraction practices.

Regarding chemical methods, organic solvents such as hexane and ethanol have been conventionally used, which, although reducing costs, attract criticism for their lack of environmental sustainability and potential health risks in medical use [16]. This has led to a shift towards more environmentally sustainable methods, with recent developments moving toward the use of environmentally friendly solvents, called ‘green solvents’ or supercritical fluids [39]. As seen, there are numerous pigment extraction techniques, but enzyme-assisted extraction stands out as an interesting strategy that allows much more efficient, sustainable, and pure pigment recovery [42]. Each method uses different mechanisms for cell disruption and applies different temperature and pressure conditions [41,43]. In the relentless search for improving the quality of the final product for use in medicine, biotechnological advances have been achieved through genetic engineering techniques, which involve the modification of organisms such as bacteria, yeast, or plants to produce carotenoids of higher purity and with better concentrations of greater and better quality [12]. This method typically involves the introduction of genes that encode carotenoid biosynthetic enzymes into the genome of the host organism [38]. By manipulating genetic pathways, it is possible to improve the production of specific carotenoids. The efficient extraction of carotenoids in medicine presents a significant challenge due to the complex nature of human health and the various diseases that need to be treated [12].

## 3. Carotenoids in Medicine and Therapeutic Treatment of Diseases

Numerous studies around the world have established the fundamental role of carotenoids in medicine and their prevalence in the therapeutic treatment of diseases, not only as a source of antioxidants, but also as a source of provitamin ‘A’ and as components of macular pigment, which underlines its potential in the prevention and treatment of various specific diseases [7,22,44,45,46,47,48,49]. Beyond their crucial role in ocular health, carotenoids have been associated with improving cognitive function, promoting cardiovascular wellness, and even potential anticancer properties, thus emphasizing their broader impact on human physiology [50,51]. This highlights the intricate and multifaceted nature of the functionality of carotenoid within the human body, showing its involvement in essential cellular processes and their potential as therapeutic agents. Ongoing research in this area continues to shed light on the therapeutic potential of carotenoids and their potential applications in the field of medicine.

### 3.1. Antioxidant Activity

Carotenoids play a critical role in the prevention and treatment of specific diseases, specifically in combating the harmful effects of reactive oxygen species (ROS) and reactive nitrogen species (RNS) in many pathophysiological conditions [52]. Their exceptional ability to act as potent free-radical inhibitors underscores their importance in preventing cellular damage and protecting against a spectrum of chronic diseases, including cardiovascular diseases, cancer, and neurodegenerative disorders.

The different molecular configurations of carotenoids, characterized by conjugated double bonds, give them the ability to effectively counteract the detrimental impact of oxidative stress, thus preserving cell integrity and mitigating the risk of chronic inflammatory diseases [53,54]. Carotenoids function as potent free-radical scavengers, preventing the spread of oxidative chain reactions and preventing the resulting cell damage [55]. Their multifaceted actions as singlet oxygen inhibitors and lipid peroxidation inhibitors highlight their critical role in maintaining redox homeostasis and preserving cell function, thus promoting general well-being and longevity [56,57]. In addition to their antioxidant capacity, they exert protective effects on other crucial biomolecules, including proteins and deoxyribonucleic acid (DNA), thereby reducing the progression of degenerative disorders induced by oxidative stress [58,59,60].

### 3.2. Anti-Inflammatory Effects

Carotenoids also demonstrate significant anti-inflammatory effects by targeting key signaling pathways and downregulating the expression of critical pro-inflammatory mediators implicated in various inflammatory conditions [61,62]. These potent phytochemicals exert their anti-inflammatory actions by modulating the activity of critical transcription factors, such as the “nuclear factor kappa light chain enhancer of activated B cells” (NF-κB), which serve as central regulators of the inflammatory cascade [63,64,65,66]. By attenuating nuclear translocation and DNA binding activity of NF-κB, carotenoids effectively inhibit the expression of a spectrum of pro-inflammatory cytokines, chemokines, and adhesion molecules, thus reducing the inflammatory response and alleviating the progression of inflammatory disorders [67,68,69].

The intricate interaction between carotenoids and various signaling cascades underscores their potential to mitigate chronic inflammatory diseases, such as rheumatoid arthritis and inflammatory bowel disease, characterized by aberrant immune responses and dysregulated inflammatory pathways [70,71]. Its ability to target multiple points along the inflammatory signaling axis highlights its versatility in damping the production of pro-inflammatory mediators, thus restoring immune homeostasis and improving clinical manifestations of inflammatory pathologies [72]. Furthermore, the immunomodulatory activities of carotenoids extend to the regulation of immune cell function, including the modulation of macrophage polarization, lymphocyte proliferation, and T cell differentiation, thus orchestrating a balanced immune response and promoting immune tolerance [73]. By influencing the interaction between immune cells and their microenvironment, carotenoids contribute to maintaining immune homeostasis and preventing excessive immune activation, ultimately reducing the pathogenesis of chronic inflammatory diseases [74,75].

### 3.3. Immunomodulatory Effects of Carotenoids

Carotenoids, including β-carotene and astaxanthin, exhibit multifaceted interactions with immune cells, exerting profound immunomodulatory effects that significantly impact immune cell proliferation, differentiation, and production of cytokines, chemokines, and growth factors. This fine-tunes the immune response and maintains immune homeostasis [76,77,78,79]. These bioactive compounds demonstrate a remarkable ability to modulate the functional activities of various subsets of immune cells, including lymphocytes, macrophages, and dendritic cells, orchestrating a balanced immune response and promoting immune homeostasis [80]. Due to their immunomodulatory properties, carotenoids show great promise in the management and treatment of various immune-related disorders, including autoimmune diseases and immunodeficiencies [81,82,83]. Its diverse actions include, as observed, T cell differentiation, B cell activation, and natural killer cell function, resulting in a balanced immune environment that effectively protects against aberrant and immune-mediated pathologies [84,85].

### 3.4. Carotenoids and Cardiovascular Health

Phytochemicals such as lycopene and β-carotene have attracted much attention for their crucial role in promoting heart health and mitigating the risk of cardiovascular disease (CVD) through their multifaceted biological activities [86,87]. Lycopene, found in tomatoes and their products, has emerged as a prominent cardioprotective agent, attributed to its notable antiatherogenic properties, particularly its ability to inhibit the oxidation of low-density lipoproteins (LDL), a key step in the progression of the onset and onset of the disease of atherosclerosis [88,89,90]. By preventing LDL oxidation, lycopene effectively reduces the formation of atherosclerotic plaques, thus reducing the risk of coronary artery disease and improving cardiovascular health [91,92,93]. Furthermore, β-carotene, a potent precursor of vitamin A, exerts important cardioprotective effects through its anti-inflammatory properties, thus contributing to improvements in various cardiovascular diseases [76,94,95,96]. Its conversion to vitamin ‘A’ within the body allows the modulation of immune responses and negative regulation of inflammatory mediators, thus attenuating the progression of chronic inflammatory conditions and reducing the risk of cardiovascular pathologies, including atherosclerosis and myocardial infarction [97,98].

### 3.5. Carotenoids and Implications for Visual Health

Lutein and zeaxanthin play an indispensable role in preserving visual health and preventing age-related macular degeneration (AMD), the leading cause of visual impairment and blindness in the elderly population [99,100]. Its antioxidant properties are essential to combat the harmful effects of reactive oxygen species (ROS) and mitigate cumulative damage caused by light-induced oxidative stress, thus safeguarding the integrity of the macular region and preserving visual acuity [101].

### 3.6. Carotenoids and Cancer Prevention

Several studies indicate that carotenoids also play an important role in cancer prevention, mostly through their potent chemopreventive properties, which involve the suppression of mutagenesis, reduction in oxidative stress, induction of apoptosis in cancer cells, and inhibition of angiogenesis, a critical process in tumor development and progression [102,103]. Their strong antioxidant capabilities allow them to neutralize reactive oxygen species (ROS) and curb oxidative damage, thus mitigating the risk of DNA mutations and cell abnormalities that can culminate in carcinogenesis [103,104].

#### 3.6.1. Chemopreventive Properties

Carotenoids’ chemopreventive potential is observed by inducing apoptosis in cancer cells, triggering programmed cell death, and preventing the uncontrolled proliferation of malignant cells, thereby stopping tumor progression and metastasis spread [105,106,107]. Its ability to modulate signaling pathways and regulatory mechanisms involved in cell growth and survival underlines its fundamental role in preventing the onset and progression of various malignancies [108,109]. Furthermore, the ability of carotenoids to suppress angiogenesis, a critical step in tumor progression, underscores their potential to prevent tumor growth and metastasis [110,111,112]. Their regulatory influence on the formation of new blood vessels and their ability to alter the microenvironment leading to tumor expansion exemplifies their multifaceted role in preventing the angiogenic cascade, which is critical for limiting tumor development and reducing the spread of cancer cells [110,113].

#### 3.6.2. Epidemiological and Clinical Evidence

A large body of epidemiological and clinical evidence underscores the important role of carotenoids in cancer prevention, particularly in reducing the risk of lung, breast, and prostate cancer. Different studies have established a strong correlation between a high dietary intake of carotenoids and a reduced susceptibility to these prevalent forms of cancer [114,115]. Specifically, diets rich in β-carotene and lycopene have shown strong protective effects against the development and progression of these malignancies, thus highlighting the vital role of these bioactive compounds in preventing cancer incidence [116,117]. The documented association between higher carotenoid consumption and lower risk of lung cancer underscores the important potential of these compounds to mitigate the detrimental impact of various carcinogens on lung tissue [118,119,120]. Furthermore, the protective influence of carotenoids against breast cancer has been elucidated through extensive clinical research, shedding light on their critical role in modulating hormonal imbalances and reducing cellular aberrations that can precipitate breast carcinogenesis [121,122]. On the other hand, there is compelling clinical evidence for the preventive properties of carotenoids against prostate cancer, highlighting their potential as critical agents to stop the onset and progression of this prevalent malignant disease [123,124,125]. Its ability to modulate cell signaling pathways and prevent uncontrolled proliferation of prostate cancer cells underscores its important role in reducing the incidence and burden of this widespread form of cancer [126,127,128].

### 3.7. Emerging Applications in Medicine

As science advances, carotenoids are increasingly recognized for their emerging applications in therapeutic medicine, particularly in the realms of wound healing and metabolic health [129]. Through their multifaceted properties, specific carotenoids such as β-carotene and astaxanthin have shown promising wound healing attributes by orchestrating modulation of inflammatory responses and oxidative stress [130]. Their role in accelerating tissue repair and regeneration has shown the potential to improve wound closure rates and minimize scar formation, underscoring their importance as key therapeutic agents in the treatment of various wound-related complications [131,132]. Furthermore, the distinctive contributions of lutein and zeaxanthin to metabolic health have attracted significant attention due to their potential to improve insulin sensitivity and regulate glucose metabolism [29,133]. Their documented influence in reducing markers associated with metabolic syndrome, a complex interplay of metabolic abnormalities related to increased cardiovascular risk, underscores their critical role in promoting metabolic homeostasis and protecting against the progression of metabolic disorders [134,135,136].

### 3.8. Carotenoids and Nanotechnology

The use of nanotechnology in the formulation of carotenoids represents a significant advance in the fields of nutritional and pharmaceutical sciences and emerges as a promising avenue to improve the bioavailability and non-degradation of active carotenoid compounds, which are essential nutritional components essential with important health benefits [137,138]. By reducing carotenoid particles to nanometer sizes, their absorption and availability within the body can be significantly improved [139,140]. This approach offers a potential solution to the long-standing challenge of poor bioactivity and bioavailability associated with conventional carotenoid formulations [141]. Studies have shown that nanosized carotenoid particles, typically in the range of a few nanometers, exhibit greater solubility and stability, allowing easier absorption in the gastrointestinal tract [142]. The smaller particle size allows for better dispensability and interaction with body cells, facilitating more efficient utilization [143]. Furthermore, the higher surface-to-volume ratio of these nanosized particles promotes a better interaction with biological membranes, leading to greater bioavailability and greater tissue accumulation [137,144,145]. Thanks to continued advances in R&D, nanosized carotenoid particles have the potential to revolutionize the delivery and efficacy of these essential dietary compounds, contributing to better health outcomes and more precise and timely disease prevention [142].

Various techniques, such as supergravity rotating packed-bed crystallization, have been developed to produce nanodisperse microcapsules with a high content of E-carotenoid, ensuring a high content of transisomers and optimal bioactivity and bioavailability [146,147]. This innovative approach not only improves carotenoids but also contributes to their sustained release over time, resulting in longer and more effective biological activity. There is already a documented and patented process for producing microcapsules with a high E-carotenoid content [148]. In parallel, work is underway on new encapsulation techniques, with gelatin-based protective colloids that allow greater stability and prevent capsule breakage. This is mainly due to the instability of carotenoids, especially with respect to heat treatments and oxygen sensitivity [149,150,151,152].

#### Supramolecular Carriers

Supramolecular transporters, also known as supramolecular carriers, are advanced delivery systems designed to improve the absorption and bioavailability of various compounds, including carotenoids [153,154]. These transporters use principles of supramolecular chemistry, which involves the assembly of molecules through noncovalent interactions, to improve the solubility, stability, bioavailability, and cellular uptake of carotenoids in humans. These advanced delivery systems protect carotenoids from degradation, facilitate their absorption by intestinal cells, and provide controlled release, ensuring that the body effectively absorbed and utilizes carotenoids [155,156,157]. The fundamental property of supramolecular transporters lies in their ability to alter their surface by introducing additional functional groups, facilitating targeted delivery to specific organs or cellular receptors. The formulation of functional nanostructured carriers allows the release of drugs as needed in specific microscopic environments associated with various diseases [158,159]. The same authors point out that nanostructured carriers include lipid nanoparticles, polymers, micelles, inorganic nanoparticles, hybrid nanoparticles, and other types of particles. Applications of supramolecular transporters have been used in various domains, including biomedicine and materials science [160,161]. Their unique optical and electronic properties make them promising candidates for use in sensors, biomaterials, and optoelectronic devices, offering interesting perspectives for the development of advanced technological solutions [162,163]. Table 3 shows a more detailed explanation of the mechanisms, how supramolecular transporters improve carotenoids absorption in humans, their advantages, some examples and potential applications.

## 4. Innovation and Technological Advances in Carotenoids

Innovation in carotenoid biosynthesis, metabolic pathways, regulatory mechanisms, and bioavailability of biomolecules are essential to develop efficient production processes that obtain high-quality carotenoid-based compounds specific for specific diseases [164]. In the medical sector, the continuous generation of knowledge related to carotenoids drives innovation in extraction methodologies, purification techniques, and biotechnological approaches, leading to higher product yields, greater bioavailability, traceability, specificity, and fundamentally better preventive and curative actions [165].

The dynamic nature of innovation generation in the carotenoid production sector encourages the development of new drug formulations and delivery systems that improve the bioactivity, stability, solubility, and targeted delivery of carotenoid-based pharmaceuticals [166]. In the same vein, the integration of interdisciplinary research efforts promotes a deeper understanding of the interactions between carotenoids and cellular processes, thus providing information on their mechanisms of action and their possible applications in the prevention and treatment of diseases [167]. Collaborative knowledge exchange between researchers, biochemists, pharmacologists, physicians, nanotechnologists, bioinformatics specialists, artificial intelligence specialists and biotechnologists plays a critical role in accelerating the development of innovative strategies for the production, purification, and formulation of high-impact carotenoids in medicine [168,169]. A comprehensive understanding of carotenoids factors that influence the bioactivity, bioavailability, stability, and pharmacokinetics allows the design of optimized drug delivery systems that improve their therapeutic efficacy and minimize adverse effects, as seen in the case of supramolecular carriers [170,171].

Generating innovative products is crucial in the context of the production of carotenoids for medicinal purposes. It requires a deep understanding of its biosynthetic pathways, cutting-edge extraction techniques related to biotechnological approaches, and genetic engineering techniques aimed at improving the production efficiency, purity, and bioavailability of the compounds [164,165]. Knowledge about the intricate biochemical processes involved in carotenoid biosynthesis is essential in developing cutting-edge production strategies that ensure high yields and superior product quality [157]. Advances in bioprocessing technologies and fermentation techniques have facilitated the scalable and cost-effective production of carotenoids, enabling their widespread application in pharmaceutical formulations and nutraceuticals [172,173].

Furthermore, the integration of analytical techniques and quality-control measures is vital to ensure the safety, efficacy and stability of carotenoid-based medications [174]. The use of innovative delivery systems, such as nanoformulations and liposomal carriers, has revolutionized the field of carotenoid therapy, allowing targeted delivery and increased bioavailability of these bioactive compounds [166]. A comprehensive understanding of the biological mechanisms underlying the therapeutic effects of carotenoids, together with innovative research efforts and cooperation, may pave the way for the development of new pharmacotherapies and preventive interventions, thus addressing the growing global burden of chronic diseases [175,176].

### The Dissemination of New Carotenoid Development Techniques

Different methods of carotenoid extraction are essential for the subsequent use of this type of molecule. However, there are numerous facets related to the quality of the carotenoid obtained and that have to do with its purity and which are important to address. Along these lines, we can say that there are different techniques, already used in other nearby industries, such as biopharmaceuticals, that could be of great help in the quality control of carotenoids obtained for medical purposes related to the purity of the carotenoid obtained.

To improve the analytical rigor and guarantee constant quality of the carotenoid, there are, among others, two important methods to consider, the one referring to advanced quality by design (QbD) and multiple attribute methods (MAM) [177,178]. The application of QbD in analytical sciences, particularly in method development, involves systematic approaches such as ‘Design of experiments’ (DoE) and risk assessments to optimize analytical performance and ensure the robustness of the results [179,180,181]. These principles guide the establishment of the ‘Method Operable Design Region’ (MODR), essential to achieve the desired quality in the measurement of specific properties of carotenoids. Furthermore, the integration of QbD into the development phases supports the regulatory flexibility and improves methodological consistency, reducing the variability in the results [182]. Furthermore, MAMs offer significant advantages in carotenoid analysis, providing a comprehensive and integrative approach to study various attributes simultaneously [183]. These methods would enable the accurate and synchronous quantification of multiple carotenoids in complex matrices, improving both efficiency and analytical performance. This is particularly beneficial in strict regulatory environments such as those of the Food and Drug Administration, where accurate evaluation of carotenoid concentrations in health products is the key to approval [184]. In addition, MAM methods facilitate the integration of multiomics data, providing information on the interactions between carotenoids and other biomolecules, thus improving the understanding of their biological functions and effects on lipid profiles [185,186]. MAMs also contribute to the improvement of carotenoid analysis by using different methods such as HPLC combined with MS and DAD, UV and visible spectrophotometry, thin-layer chromatography (TLC), and supercritical fluid chromatography, offering a robust approach to accurate identification and quantification of carotenoids even in the presence of other similar compounds [187,188]. Advanced MS technologies, such as ultra high-performance liquid chromatography–MS (UHPLC-MS) and data-independent MS (MSE) workflows, improve the detection and characterization of carotenoids in complex biological matrices [189]. These methods allow the precise quantification of hormones derived from carotenoids and apocarotenoids (APOs) from plant tissues, helping to understand their metabolism and regulatory functions [18,190].

The use of high-resolution hybrid mass spectrometers coupled with HPLC enables the precise determination of carotenoid profiles in plant tissues and food samples, generating valuable data to unravel complex results [191,192]. The time required to perform HPLC analysis of carotenoids has been drastically reduced [193,194,195]. This is due to a number of factors, such as smaller-particle-size columns operating at lower pressures, the development of isocratic methods, and more recently, the use of a C30 column [196]. The C30 column is capable of isocratically separating a wider range of carotenoids, eliminating the need to run a gradient and, therefore, reducing the analysis time [197]. Another development is the production of superficially porous particles (SPP) that have an efficiency similar to that of small particles but use lower pressures [198]. Although SPP columns have not yet been tested for carotenoids, they are likely to become the column of choice due to their efficiency and cost-effectiveness. Because of these advances, it appears that the choice of column and the time it takes to run the analysis would no longer be a limiting factor for carotenoid HPLC.

However, the implementation of MAMs for the analysis of carotenoids is not always optimal, as it also presents some technological challenges that can disturb the results in some specific cases. A very important one is the possible interference of complex biological matrices, which can hinder the detection of carotenoids, requiring advanced purification and isolation techniques with additional costs and time [199,200]. Furthermore, the structural characterization of carotenoid metabolites requires robust analytical protocols and equipment parameters, as even minor deviations can lead to significant errors in their quantification [201]. However, there are different technologies for overcoming biological interferences in carotenoid analysis.

Traditional methods to assess carotenoid bioavailability have provided valuable information, but recent technological advances have paved the way for innovative approaches that offer more detailed and accurate evaluations. These cutting-edge methodologies range from in vitro models and advanced analytical techniques to in vivo studies and novel in silico tools. They also include emerging technologies such as single-cell transcriptomics, CRISPR-based gene editing, and microfluidics-based gut-on-a-chip systems, which together are transforming our understanding of how carotenoids are processed in the body [202]. These innovations not only improve our knowledge of carotenoid bioavailability, but also have the potential to inform the development of more effective nutritional interventions and supplements. Table 4 highlights the key aspects of different innovative methods, including their contributions to the characterization of carotenoid bioavailability, their specific advantages and the techniques involved.

## 5. Discussion

This research presents an analysis related to innovative formulations for the development of new drugs and combination therapies that incorporate different carotenoids in medicine and in the treatment of diseases. Recent research findings on carotenoids in medicine have focused on their metabolism, the use of biotechnology, and their nutritional and health benefits. Their consumption has been associated with a reduced risk of vitamin A deficiency, age-related macular degeneration, cancer, and cardiovascular and skin diseases [99,213,214,215]. Significant progress has been made in understanding the bioavailability of carotenoids and their potential use in the cosmetic industry and skin-related diseases [216,217]. The close integration of knowledge and innovation in the production of carotenoids for medicinal applications has the potential to drive advances in personalized medicine and precision healthcare [218]. In other words, advances in the knowledge of carotenoids in the prevention and treatment of diseases underscore their potential as indispensable therapeutic agents, capable of mitigating the pathological consequences of oxidative stress and inflammation, thus offering promising perspectives for the development of new strategies in the therapy and improvement of chronic diseases [219,220,221,222].

Comprehensive research on how carotenoids modulate the immune system highlights their critical role in shaping immune cell function and orchestrating a balanced immune response, indicating their therapeutic potential in the management and treatment of various blood-related disorders of the immune system [95]. A comprehensive understanding of the anti-inflammatory effects produced by carotenoids underlines their crucial role in modulating inflammatory signaling pathways and regulating immune responses, thus offering promising perspectives for the development of new therapeutic interventions in the management and treatment of a wide range of inflammatory disorders [71]. The influence of carotenoids on the proliferation and differentiation of immune cells further enhances their potential to promote immune tolerance and improve severity cases related to autoimmune diseases characterized by dysregulated immune responses and altered immune tolerance [223,224]. Their multifaceted contributions to redox signaling pathways, immune modulation, and inflammatory responses highlight their critical role in orchestrating cellular homeostasis and strengthening defense mechanisms against a variety of diseases [225,226]. The multifaceted chemopreventive attributes of carotenoids enhance their potential as valuable therapeutic agents in cancer prevention, emphasizing the importance of their incorporation into diet regimens and their integration into new therapeutic strategies that aim to curb the incidence and progression of various malignancies [227,228]. The accumulation of epidemiological and clinical evidence supporting the cancer prevention attributes of carotenoids serves as a compelling impetus to integrate carotenoid rich diets and supplementation strategies into the prevention and management regimens of certain cancers, highlighting their potential as therapeutic agents in the fight against cancer [229]. The accumulation of evidence also highlights the benefits of carotenoids in some emerging applications in medicine, such as wound healing and metabolic health, where it emphasizes their enormous potential as valuable therapeutic adjuncts to address a variety of complex medical conditions [230,231].

This study also emphasizes the importance of research on the use of carotenoids, particularly lutein. This compound is of particular interest due to its demonstrated effectiveness in minimizing the progression of age-related macular degeneration and cataracts, highlighting the importance of promoting its consumption to maintain adequate ocular health [49,232]. Its role as an antioxidant and blue-light filter, which amplifies its concentration in the macula lutea of the retina, is key. This presence significantly reduces chromatic aberration and serves as a defense against light-induced oxidative stress [233,234]. Second, specific carotenoids, such as β-carotene, serve as precursors of vital retinoids and participate in critical processes such as retinoid cycling within the eye [235,236,237]. These retinoids are essential in several processes, including retinoid cycling within the eye, where visual pigments facilitate phototransduction. These pigments, closely aligned with G protein-coupled receptors, play a critical role in the translation of light into nerve signals [238,239]. Furthermore, retinoid oxidation leads to the synthesis of all-trans retinoic acid (RA), a vital substance that binds to retinoic acid receptors (RAR) to influence gene expression in a broad spectrum of physiological processes [240,241,242]. This underscores the imperative need to incorporate these potent carotenoids into dietary regimens and therapeutic interventions aimed at promoting ocular well-being and mitigating the risk of vision-related disorders [243]. By acting as a protective barrier against blue light and oxidative stress, these carotenoids maintain the structural and functional integrity of photoreceptor cells, thus preserving optimal visual function and improving overall ocular health [244,245].

Scientific research has made significant progress in the prevention of cardiovascular diseases through concerted actions of lycopene and β-carotene, underscoring their fundamental role in mitigating the risk of cardiovascular events (CVD) and reducing the progression of cardiovascular disease atherosclerosis through its synergistic antiatherogenic and anti-inflammatory properties [246,247]. These profoundly bioactive compounds offer promising prospects for developing innovative therapeutic strategies aimed at preventing and treating many cardiovascular disorders, highlighting their importance in promoting general cardiovascular well-being [248].

A crucial point concerns the extraction of carotenoids. This is key in estimating the quality and traceability of the origin of these compounds, depending on the needs sought in the different fields of medicine. As we have seen, this could be due to unsustainable chemicals or environmentally friendly solvents. However, genetic engineering offers the best advantage of producing carotenoids with high purity and structural characteristics similar to those found in nature, with better impact and optimal results in combating specific diseases and at a reasonable cost compared to synthetic origins [249]. The extraction of carotenoids is essential not only because of the way they are incorporated into nutritional and medical products and into the body itself. In this sense, it is imperative to improve the interpretation of the data reported on the net content of carotenoids after the different technological treatments to which they are subjected, in addition to establishing appropriate calculation formulas for each specific case [95]. Therefore, the contributions of other studies that have deepened the structural knowledge and properties of supramolecular carriers are important, highlighting their various self-assembly mechanisms and molecular architectures [250]. In this regard, innovative strategies have been employed to design and synthesize increasingly complex supramolecular carriers, leading to a better understanding of their unique properties and functionalities [74,157,251]. Collaborative efforts between supramolecular chemists and carotenoid researchers will be crucial to unlocking the full potential of supramolecular carotenoids to address complex technological challenges related to therapeutics, medicine, and the prevention and treatment of complex diseases [252].

The key to reflecting the use of carotenoids in medicine and their application in the therapeutic treatment of diseases in a highly efficient and innovative product depends on our understanding of their properties, reactions, and benefits, as well as their drawbacks at organic, molecular, technological and strategic levels, as well as their behavior in various commercial markets [29]. Continuous advancement in scientific research in the field of carotenoids and the design of new molecular architectures drives the development of new drug formulations and delivery systems that improve the stability, solubility, and targeted delivery of carotenoid-based pharmaceuticals, thus improving its effectiveness and clinical applicability [253]. This continuous progress in scientific research and innovation (R&D) in the field of carotenoids drives the emergence and refinement of new carotenoid formulations in medicine, increasing product stability and solubility, and targeting the delivery of specific carotenoid-based therapeutic drug treatments, thus improving their clinical effect, efficacy, and applicability in the treatment of specific diseases [166].

This research highlights a growing emphasis on the development of carotenoid-rich formulations for the treatment of age-related macular degeneration, cognitive disorders, oncology treatments, and other chronic diseases, highlighting their importance in contemporary medical research.

A major challenge for the future of carotenoids is related to the dynamic interaction between improving their bioavailability and adopting personalized approaches to carotenoid consumption, exemplifying the evolving landscape of medical applications. In this sense, the integration of MS into multiattribute methods would significantly improve the identification, quantification, and characterization of carotenoids, essential for studying their functions and metabolic pathways. This integration allows carotenoids from complex backgrounds without interference from other substances, ensuring more accurate measurements. Furthermore, the application of techniques such as matrix solid-phase dispersion (MSPD) further exemplifies how MAM could optimize carotenoid preparation and extraction processes, leading to more efficient analysis workflows [254]. These methods have been crucial in determining the content of carotenoids in different food products, ensuring precision and reliability in the evaluation of the presence of carotenoids such as lutein and zeaxanthin in supplements and food products. These methodologies not only reduce the sample preparation time, but also increase throughput, which ultimately makes it feasible to handle multiple samples simultaneously and more efficiently [255]. As can be seen, MAMs in the analysis of carotenoids use several technologies to overcome biological interference. Techniques such as linked scanning mass spectrometry help identify carotenoid compounds accurately, even in the presence of contamination [256]. For isolation and identification, methods that involve protection from light, heat, and oxygen are crucial to prevent degradation and maintain integrity [257]. The use of online supercritical fluid extraction in conjunction with supercritical fluid chromatography and mass spectrometry enables an efficient and accurate analysis of carotenoids and their cleavage products, reducing extraction time and improving analytical precision [258,259].

Using strategies aimed at maximizing carotenoid absorption and tailoring therapeutic regimens based on individual genetic and health profiles, the scope of carotenoid-based interventions continues to expand, fostering new avenues for precise and effective healthcare delivery along with the increasing popularity of artificial intelligence. The diverse applications of carotenoids in medicine extend to their role in improving bioavailability and the potential for personalized approaches, thus contributing to the burgeoning field of personalized medicine. Given the variability in carotenoid bioavailability influenced by bioactivity, individual genetic predispositions, and dietary patterns, strategies aimed at increasing their bioavailability have gained importance. Incorporating dietary fats along with carotenoid-rich foods and using innovative delivery systems, such as nanoparticles or emulsions, are recognized as effective strategies to improve the absorption and bioavailability of carotenoids, thereby maximizing their therapeutic potential.

**Figure 1:**
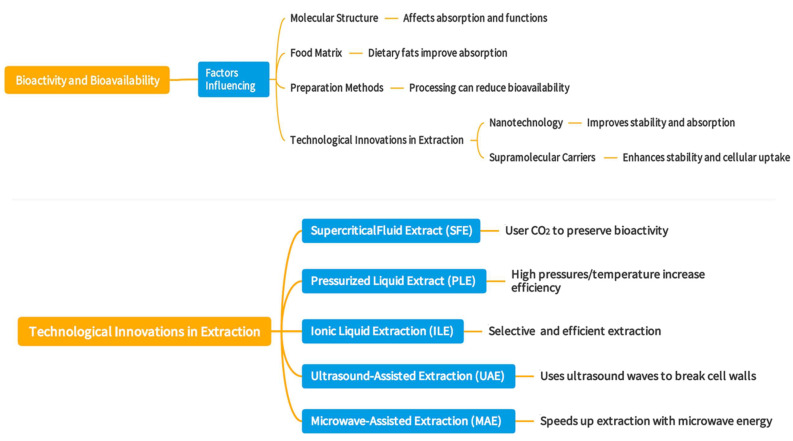
Overview of carotenoids related to their bioactivity, bioavailability, and technological extractions.

**Figure 2:**
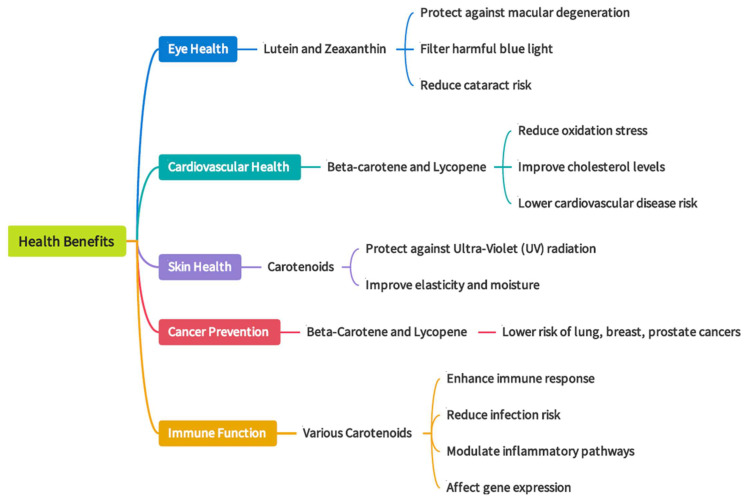
Overview of carotenoids related to their benefits for human health.

Table 5 summarizes the topics analyzed in this investigation and some of the highlighted references for each of the identified grouped keywords, while Figure 1 and Figure 2 show a complementary and more detailed view of carotenoids based on their bioactivity, bioavailability, different extraction methods, and the most important benefits for human health.

## 6. Conclusions

**Scheme 1:**
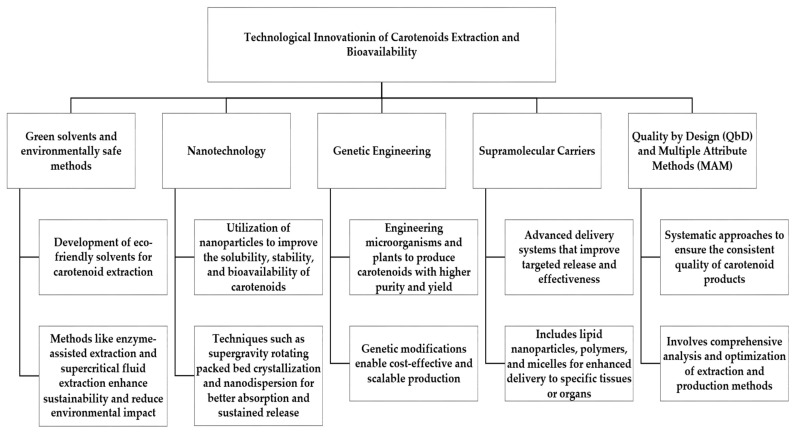
Different modes of technological innovation in carotenoid extraction and bioavailability.

**Scheme 2:**
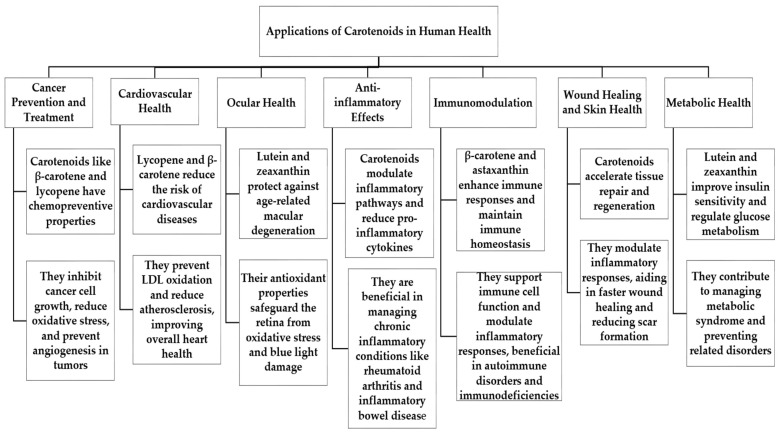
Applications of carotenoids in human health (cancer prevention and treatment; cardiovascular health; ocular health; anti-inflammatory effects; immunomodulation; skin health; and metabolic health).

This research presents a detailed and methodical assessment of the role and applications of carotenoids in therapeutic medicine, highlighting their fundamental role in the treatment of a variety of diseases. This analysis synthesizes the findings of an extensive review and offers a comprehensive perspective on the multifunctional nature of carotenoids and their emerging applications in medical science. Scheme 1 and Scheme 2 summarize the most notable elements related to the findings of this research. Scheme 1 shows some technologies related to the extraction methods and bioavailability of carotenoids used in human health. On the other hand, the innovative applications of carotenoids in human health can be seen in Scheme 2. A detailed explanation of the Scheme 1 and Scheme 2 is given in the following.

Biological bioactivity and therapeutic potential: Carotenoids, mainly synthesized by phototrophs, bacteria, and fungi, are identified by their antioxidant, anti-inflammatory properties, and provitamin A activity. These pigments, divided into carotenes and xanthophylls, are essential for the maintenance of human health and influence eye health, cardiovascular health, skin health, immune and cognitive functions, and bone health.

Mechanisms of action: Research looks at the mechanisms through which carotenoids exert their effects. They neutralize free radicals, modulate gene expression, and regulate inflammatory pathways. This multifaceted action makes them effective against chronic diseases such as cancer, cardiovascular disease, and age-related macular degeneration.

Advances in carotenoid research: This study highlights significant advances in the understanding of carotenoid metabolism, absorption, and bioavailability, particularly through the advancement of supramolecular transporters. These advances have opened new avenues to improve the therapeutic efficacy of carotenoids.

Application in medicine: We emphasize the growing recognition of carotenoids in medicine, particularly in wound healing and metabolic health. Specific carotenoids, such as β-carotene and astaxanthin, have shown promising attributes in wound healing by modulating inflammatory responses and oxidative stress. The distinctive contributions of lutein and zeaxanthin to metabolic health have attracted significant attention, illustrating their potential to improve insulin sensitivity and regulate glucose metabolism.

Nanotechnology and carotenoids: The use of nanotechnology in the formulation of carotenoids is a significant advance in the nutritional and pharmaceutical sciences. It addresses the long-standing challenge of poor carotenoid bioavailability by demonstrating that carotenoid nanoparticles exhibit increased solubility, stability, and absorption, enhancing their therapeutic potential.

Finally, this study meticulously illustrates the intricate interplay between the bioactive properties of carotenoids and their applications in medicine. It reveals their growing potential in disease prevention and treatment, guided by ongoing scientific research and innovation. The interconnection of carotenoids with various cellular processes and their potential as therapeutic agents is evident. Continued innovation with the use of cutting-edge technologies in this field is essential to advance the medical applications of carotenoids, which contribute significantly to the field of precision healthcare and personalized medicine

## 7. Future Directions

Carotenoids, colorful pigments abundant in fruits and vegetables, are poised to play an even greater role in the promotion of human health in the coming years. Here is a closer look at some of the exciting research directions:

- Targeting Chronic Diseases: Researchers are excited about the potential of carotenoids to combat a variety of chronic health issues. Studies are exploring their effectiveness in preventing and managing conditions such as metabolic disorders (think diabetes), cardiovascular diseases (heart disease and stroke), and even neurodegenerative disorders (Alzheimer’s and Parkinson’s).
- Unlocking Gut Microbiome Connections: A fascinating new area of research is investigating the link between gut bacteria and how our bodies process carotenoids. Understanding how these tiny intestinal residents influence carotenoid metabolism and health effects can pave the way for personalized approaches. Imagine creating dietary or supplement plans tailored to an individual’s gut microbiome to maximize the benefits they receive from carotenoids!
- Engineering Carotenoids for Enhanced Health: The future might involve not just consuming naturally occurring carotenoids, but also potentially creating new ones with even greater health benefits. Genetic engineering holds promise for modifying plants to produce higher levels of existing carotenoids or even creating entirely new varieties with specific health-promoting properties.
- Beyond Disease Prevention: Carotenoids are not just disease fighters. Research is also investigating their broader roles in plant health and function. This deeper understanding could lead to advancements in agriculture, allowing us to cultivate crops with enhanced nutritional value and resilience.
- Advanced Detection Methods: Imagine a non-invasive way to measure your body’s carotenoid levels! Techniques such as Raman spectroscopy are being explored for their potential to provide real-time information on carotenoid status. This could be a game-changer for personalized nutrition plans, allowing adjustments based on individual needs.

In essence, the future of carotenoid research is bursting with possibilities. By exploring their potential to fight disease, personalizing their benefits through connections with the gut microbiome, and developing new production and detection methods, carotenoids hold the key to unlocking a new era of proactive health management.

## Figures and Tables

**Table 1:** Specificity of the distinctive molecular structures of seven different carotenoids.

Carotenoid	Molecular Structure	Specific Features	Specificity
Beta-Carotene	C_40_H_56_	11 conjugated double bonds, cyclic end groups	Vitamin A precursor, antioxidant
Lycopene	C_40_H_56_	11 conjugated double bonds, linear structure, no cyclic end groups	Non-provitamin A, antioxidant
Lutein	C_40_H_56_O_2_	10 conjugated double bonds, hydroxyl groups on terminal rings	Eye health, antioxidant
Zeaxanthin	C_40_H_56_O_2_	10 conjugated double bonds, hydroxyl groups on terminal rings	Eye health, antioxidant
Alpha-Carotene	C_40_H_56_	Similar to beta-carotene, different double bond arrangement	Vitamin A precursor (less efficient), antioxidant
Beta-Cryptoxanthin	C_40_H_56_O	9 conjugated double bonds, one hydroxyl group	Vitamin A precursor, antioxidant
Astaxanthin	C_40_H_52_O_4_	13 conjugated double bonds, keto groups on terminal rings	Non-provitamin A, strong antioxidant, benefits for skin health, inflammation, and immune function

**Table 2:** Summary of the steps, samples, methods, and optimization.

Sample	Method	Optimization
Sample Preparation	Homogenization, Cryogenic grinding	Use of low temperatures and proper homogenization techniques to preserve carotenoid integrity.
Extraction	Solvent extraction, Supercritical fluid extraction (SFE), Ultrasound-assisted extraction (UAE)	Optimization of solvent type, pressure, temperature, and ultrasonic power to enhance extraction.
Separation	high-performance liquid chromatography (HPLC), Ultra high-performance liquid chromatography (UHPLC)	Selection of appropriate columns, mobile phases, and temperature control for high resolution separation.
Detection	UV-Vis Spectrophotometry, Mmass spectrometry (MS), Diode array detection (DAD)	Use of advanced detection techniques to accurately identify and quantify carotenoids.
Quantification	External and Internal Standard Methods	Comparison of sample results with known standards for precise quantification.

**Table 3:** How do supramolecular transporters improve the absorption of carotenoids in humans?

Characteristic	Mechanism	Advantages	Examples of Carotenoids	Applications
Enhanced Solubility and Dispersibility	Micelles: The hydrophobic core and hydrophilic exterior solubilize carotenoids.	Improved solubility in aqueous environments.	Beta-carotene, lutein, astaxanthin	Nutraceuticals, functional foods
	Cyclodextrins: The hydrophobic cavity and the hydrophilic outer surface form host-guest complexes.	Increased solubility of water.	Beta-carotene, lycopene	Dietary supplements, food fortification
	Liposomes: The lipid bilayer encapsulates carotenoids.	Enhanced dispersibility in gastrointestinal fluids.	Astaxanthin, lutein	Pharmaceuticals, cosmetic formulations
Increased Stability and Protection	Encapsulation: Protect carotenoids from oxidative degradation, light, and heat.	Maintain bioactivity during processing, storage, and digestion.	Beta-carotene, lycopene, astaxanthin	Nutraceuticals, pharmaceuticals
	Antioxidant Coencapsulation: Additional protection against oxidative damage.	Further, it improves the stability of carotenoids.	Various carotenoids	Dietary supplements, functional foods
Enhanced Cellular Uptake	Size and Surface Modification: Optimal size for endocytosis, surface ligands for targeting.	Increased efficiency of intestinal cell uptake.	Beta-carotene, lutein, astaxanthin	Pharmaceuticals, nutraceuticals
	Targeting Ligands: Enhanced specificity for intestinal cells.	Improved targeted delivery and absorption.	Lutein, zeaxanthin	Eye health supplements, pharmaceuticals
Controlled Release Profiles	pH-Responsive Carriers: Carotenoids release in response to gastrointestinal pH.	Sustained and controlled carotenoid release.	Beta-carotene, lutein	Pharmaceuticals, nutraceuticals
	Enzyme-Responsive Carriers: Release upon exposure to specific gastrointestinal enzymes.	Ensures gradual release and absorption.	Various carotenoids	Dietary supplements, functional foods
Overcoming Biological Barriers	Mucus Penetration: Nanocarriers penetrate the mucus layer in the intestinal tract.	Enhanced interaction with intestinal epithelial cells.	Various carotenoids	Pharmaceuticals, nutraceuticals
	Paracellular transport: Temporary opening of tight junctions between epithelial cells.	Facilitates the passage of carotenoids through the paracellular route.	Various carotenoids	Pharmaceuticals, functional foods
Interaction with Lipid Absorption Pathways	Chylomicron Formation: Lipid-based carriers incorporated into chylomicrons.	Efficient transport of carotenoids through lipid transport mechanisms.	Beta-carotene, astaxanthin	Nutraceuticals, functional foods
	Lipid Transport Proteins: Interaction with transport proteins such as SR-BI.	Facilitates carotenoid uptake and transport.	Various carotenoids	Pharmaceuticals, nutraceuticals

**Table 4:** The innovative methods for characterizing carotenoid bioavailability.

Method	Innovation	Advantages	Techniques
Single-Cell Transcriptomics [203]	Study of metabolism at individual cell level	High-resolution data on cell-specific responses	Drop-seq, 10× Genomics
CRISPR/Cas9 Gene Editing [202]	Precise genetic modifications	Identifies specific genes affecting bioavailability	CRISPR Knockout Models
Organoid Cultures [204]	3D miniaturized organ models	Mimics human intestine closely	Gut Organoids
Microfluidics-Based Gut-on-a-Chip [205]	Simulates human gut environment	Real-time observation in controlled setting	Organ-on-Chip Devices
Super-Resolution Imaging [206]	Visualization at nanometer scale	Detailed carotenoid distribution within cells	STED Microscopy
Metabolic Flux Analysis [207]	Measures metabolic reaction rates	Dynamic view of carotenoid metabolism	Stable Isotope Labeling
Multi-Omics Integration [208]	Combines genomics, proteomics, and metabolomics	Comprehensive biological insights	Integrative Analysis Platforms
Spatial Transcriptomics [209]	Maps gene expression in tissue context	Spatially resolved insights in gut	Spatially Resolved RNA Sequencing
Synthetic Biology Approaches [210]	Engineering of microbes or cells	Precise control over metabolic processes	Engineered Probiotics
Bioprinting of Tissue Models [211]	3D bioprinting for complex tissue structures	Accurate tissue models for absorption studies	3D Bioprinting
AI and Machine Learning [212]	Predicts bioavailability from complex data	Enhances data analysis and pattern recognition	Predictive Modeling

**Table 5:** Comprehensive analysis of grouped keywords; topic summary; outstanding references in the use of carotenoids in therapeutics and medicine for diseases.

Gruped Keywords	Topic Summary	Featured References
1. Carotenoids. Anti-inflammatory; Cancer and Chemopreventive Functions	Carotenoids, by modulating inflammatory pathways and regulating immune responses, show promise in treating inflammatory disorders. Additionally, their chemopreventive attributes position them as valuable agents in cancer prevention and treatment, urging their incorporation into diets.	[2,3,4,5,6,22,23,24,28,50,51,61,62,63,64,65,66,71,76,94,95,96,99,102,103,104,105,106,107,113,114,115,116,117,118,119,120,121,122,123,124,125,126,127,128,201,213,214,215,227,228,246,247]
2. Eye Health and Cardiovascular Well-being	Specific carotenoids like lutein and zeaxanthin are proven to slow age-related macular degeneration and cataract progression, emphasizing their consumption for visual health preservation. Moreover, lycopene and beta-carotene’s combined actions contribute to cardiovascular health by reducing cardiovascular disease risk and atherosclerosis progression.	[12,13,14,50,51,76,86,87,91,92,93,94,95,96,97,98,99,134,135,136,213,215,235,236,237,238,239,246,247,248]
3. Supramolecular Carriers and Applications in Medicine	Recent studies on supramolecular carriers shed light on their self-assembly mechanisms, suggesting potential collaboration between supramolecular chemists and carotenoid researchers. Carotenoids’ multifaceted applications in wound healing and metabolic health underline their significance in addressing various complex medical conditions, hinting at their potential to revolutionize medical interventions.	[29,71,95,129,130,131,132,133,134,135,136,153,154,155,156,157,158,159,160,161,162,163,168,169,170,171,172,173,174,175,176,218,219,220,221,222,230,231,250,251,252]
